# KerSpecGen: Co-piloting formal Kernel specification synthesis with refined knowledge graphs and large language models

**DOI:** 10.1371/journal.pone.0338821

**Published:** 2025-12-31

**Authors:** Zi Wang, Xiaoyu Zhu, Hongqiang Wang, Yichun Yu, Yuqing Lan

**Affiliations:** 1 School of Cyber Science and Technology, Beihang University, Beijing, China; 2 School of Software, Beihang University, Beijing, China; 3 School of Computer Science and Engineering, Beihang University, Beijing, China; Dalian Maritime University, CHINA

## Abstract

Formal verification ensures software correctness but faces challenges in kernel specification writing, which is labor-intensive, expertise-dependent, and limited to specific targets. For complex microkernels like seL4, these issues significantly reduce the practicality of formal methods. To address this, we propose KerSpecGen, a Large Language Model (LLM) based framework for synthesizing formal kernel specifications, which leverages knowledge graphs to bridge requirement descriptions and specification properties.

KerSpecGen’s core design includes three key components: 1) constructing a refined knowledge graph to represent progressive specification relationships (with deduplication, property judgment, and source annotation to improve accuracy); 2) building a custom dataset to fine-tune a model that converts specification properties into code; 3) designing LLM-oriented synthesis templates to transform generated code into complete candidate specification programs.

Evaluation on our KerSpecProperty benchmark (30 seL4 function modules, 624 properties) shows KerSpecGen outperforms the Few-shot method by an average of 7.06 percentage points in BLEU-4. Specifically, for the Llama-3.1-405B model, KerSpecGen achieves better specification quality than the Few-shot method in 23 out of 30 functional modules. To our knowledge, this is the first work to generate formal specifications for complex microkernels. KerSpecGen yields high-quality reference specifications. While requiring minor adjustments for direct execution, it substantially reduces the burden of writing specifications from scratch.

## 1 Introduction

The kernel serves as the cornerstone of all software, underpinning the security and reliability of operating systems, cloud platforms, and embedded devices, which makes its security and safety a paramount concern for both industry and academia. Formal verification, as a mathematically rigorous approach to proving software correctness, is widely recognized as a critical tool to guarantee the critical attributes (e.g., memory safety, privilege isolation) of kernel systems. With the increasing complexity of modern kernels (e.g., support for multi-core concurrency, confidential computing, and heterogeneous architectures), the demand for scalable formal verification becomes more urgent than ever. However, typical kernel formal verification efforts, such as seL4 [1], CertiKOS [2], and those focusing on specific requirements (confidential computing [3,4], concurrency [5,6], memory access [7]), all share a common, industry-wide bottleneck: low efficiency and prohibitive difficulty in specification generation.

The formal verification process relies heavily on high-quality specifications (e.g., preconditions, postconditions, invariants) that define “what the kernel should do.” Drafting these specifications requires deep domain expertise: specialists must first identify critical kernel properties such as information flow security, translate them into formal logic, and refine them to avoid ambiguity. All of these steps are labor-intensive and error-prone. To illustrate this difficulty, consider a fundamental kernel function in seL4: seL4_TCB_Create. This function creates a new Thread Control Block (TCB) for user processes and initializes its execution context, including stack, register state, and access permissions. For formal verification, the specification of seL4_TCB_Create must address at least three layers of complexity. First, preconditions: the caller must hold the “TCB creation” privilege (per seL4_Capability rules), the stack memory for the new TCB must be unoccupied and within the caller’s authorized address space, and the target CPU core must be idle. Second, postconditions: a new TCB object is added to the kernel’s object table with a unique ID, the TCB’s stack pointer points to the allocated memory, and the TCB’s privilege level does not exceed the caller’s (to enforce the principle of least privilege). Third, invariants: if memory allocation fails, no partial TCB object is left in the object table (to avoid resource leaks), and the caller’s existing capabilities remain unchanged (to prevent unintended privilege escalation).

Manually drafting such a specification is challenging for two key reasons. First, it requires cross-referencing with other kernel modules. For example, the precondition about “unoccupied memory” depends on the specification of seL4_Memory_Alloc, and the rule for “privilege level” ties to the seL4_Capability system. This manual coordination often leads to inconsistencies. Second, edge cases are easy to omit. For instance, if the target CPU is busy, the specification must clarify whether the function returns an error or queues the TCB. Ensuring this behavior aligns with the kernel’s scheduling logic requires exhaustive manual checking. Worse, verifying even a manually written specification’s basic correctness, such as whether it matches the kernel’s intended “least privilege” design philosophy (design intent alignment). Does the postcondition for “unique TCB ID” account for ID reuse after a TCB is destroyed (internal logical coherence)? Does the invariant for “no partial TCB” cover interrupts that occur during initialization (full path coverage)? Answering these questions requires specialists to cross-verify with the kernel’s design documentation and implementation code, adding weeks of extra effort to the verification process. Although the industry has an urgent need to apply formal verification to more mainstream kernels [8–10], inefficient manual specification writing remains the mainstream method. This inefficiency not only prolongs verification cycles (often taking months to years for a single kernel module) but also restricts formal verification to only a handful of high-stakes, small-scale kernels. As a result, it fails to meet the broader demand for secure kernel development. Thus, there is an urgent need for automatic specification synthesis to reduce human effort and enable scalable kernel verification.

Traditional research in kernel formal verification typically follows a “model first, verify later” paradigm: first model the kernel’s behavior, then use automatic theorem provers to complete verification. For example, Nelson extended traditional verifiers using symbolic execution to automatically discover errors [11], and another study extended parameterized kernel verification to automate the checking of runtime errors and privilege escalation [12]. However, these methods still require manual specification writing–making them only semi-automated. Additionally, they suffer from invariant fragility (small kernel changes break existing invariants) and state explosion, and are often tailored to specific kernel domains (e.g., only memory management), limiting their scalability to full kernels.

LLM-based approaches, which have shown promise in code generation and formalization, face complementary limitations that prevent their application to kernel specification synthesis. Existing studies have demonstrated LLM capabilities in enhancing verification automation: Wu et al. [13] used LLMs to formalize mathematical competition problems; Coser et al. [14] developed interactive refinement to improve LLM accuracy in temporal logic specifications; Wang et al. [15] proposed theorem composition for LLM-assisted proof synthesis; Wen et al. [16] combined static analysis with LLMs for C code specification generation. Nigar et al. [17] presented a deep learning system for verifying Al-Quran recitation with 97.7% accuracy. The key limitation of existing LLM-based work is that it primarily focuses on improving the precision of isolated specification snippets while neglecting the logical interdependencies inherent in large-scale kernel verification. For example, specifications for memory allocation and process scheduling in a kernel are tightly coupled (e.g., a process can only access memory it is allocated), but existing LLMs generate fragmented outputs that cannot be integrated into a coherent verification system, rendering them useless for full-kernel formalization. This is mainly because the kernel is overly complex, making it difficult to establish coding conventions, and there is limited relevant research data. Additionally, there are numerous technical terms, and the documentation and requirements often have ambiguity. These factors constrain the automated generation of formal specifications for kernels, hence the scarcity of related research.

In summary, a critical unaddressed gap exists between the comprehensive automation required for practical kernel verification and the capabilities of existing approaches. Traditional methods are semi-automated (relying on manual specs), domain-specific, and cannot scale to complex kernels. Current LLM techniques generate fragmented, logically disconnected specifications that fail to capture kernel-wide interdependencies. This gap directly hinders the practical application of formal verification to mainstream kernels: without an automated way to synthesize complete, logically consistent, and kernel-specific specifications, formal verification remains too slow, costly, and expert-dependent to be widely adopted. This is the core problem our work aims to solve.

To bridge this gap, this paper proposes KerSpecGen, a novel framework designed to synthesize complete and logically consistent kernel specifications. The main contributions of this work are as follows:

1. We establish an innovative workflow that leverages LLMs to synthesize comprehensive kernel specifications. This workflow can automatically generate accurate specification code based on high-level functional requirements (e.g., “implement secure inter-process communication”) or security properties (e.g., “no unauthorized memory access”), significantly reducing manual effort and addressing the inefficiency of traditional methods.
2. We construct a complete knowledge graph of abstract specifications for the seL4 microkernel, the largest open-source verified kernel of its kind. This graph explicitly maps high-level kernel properties (e.g., “privilege isolation”) to their concrete specification counterparts (e.g., logical axioms for kernel object ownership), demystifying kernel specification complexity and enabling LLM understanding of cross-module interdependencies.
3. We develop two high-quality domain-specific datasets for kernel specification synthesis: KerSpecProperty, a property-oriented formal specification dataset that contains 30 expert-written specs for core seL4 functions—these specs are developed by domain experts based on their practical experience, with content extracted and refined from both seL4’s official Isabelle/HOL verification artifacts and relevant publicly published papers in kernel formal verification; KerSpecCode, a standard kernel code dataset used primarily for LLM fine-tuning (with auxiliary use in result cross-validation). These two datasets not only support the result evaluation of KerSpecGen but also provide a reusable benchmark for future research on automated kernel verification.

The remainder of this paper is organized as follows. Sect 2 reviews the relevant literature on formal verification and LLM-based code generation. Sect 3 details the design of the KerSpecGen framework and its core components. Sect 4 presents our experimental evaluation, including the construction of the seL4 knowledge graph and the performance of our specification generation model. Finally, Sect 5 concludes the paper and outlines promising directions for future work.

## 2 Related work

**Formal specification generation based LLM.** LLMs provide the possibility for highly automated generation of program specifications but face several issues: First, the generated content is not precise enough. General-purpose LLMs are relatively well trained in frequently used programming languages but achieve accuracy rates of only 67.0% and 48.1% [18] for program synthesis. Compared to this, LLMs are less trained in specification languages, resulting in lower-quality output. Li et al. [19] explored extracting specification properties from high-quality documents–those with precise behavioral descriptions and minimal ambiguity, but achieving high accuracy still relies on manual annotation. SpecGen [20] can generate verifiable specifications for program fragments through interactive operator mutation. However, it is limited to very simple programs and code snippets, and continuous human interaction is required to complete verification. Specllm [21] applies large language models to specification generation for integrated circuits, yet the generated specifications remain rudimentary and lack robust evaluation. Overall, specification properties produced by current LLMs are difficult to apply effectively to complex systems. Moreover, there is still no method capable of automatically evaluating and refining specifications to consistently generate high-quality properties. Second, there is a lack of accurate focus. Current LLMs typically consist of tens to hundreds of billions of layered neurons, making their output processes uninterpretable. Recent research indicates that models suffer from middle dropout phenomena [22], paying little attention to middle parts when the input content is too long. Zhan et al. [23] encoded security specification properties to detect code fragments that violate them. However, this approacfimdh suffers from limited abstraction and reasoning capabilities; even a single property often needs to be decomposed into multiple sub-properties, making it difficult to apply to large-scale programs. Krishna et al. [24] demonstrated the potential of LLMs in drafting requirement specification descriptions, yet they remain unable to establish that LLMs can perform holistic specification design with the contextual understanding that domain experts possess. For kernels, synthesizing specifications should capture and describe as much kernel behaviour as possible. Directly adopting whole synthesis (i.e., synthesizing all specifications at once) may produce unsatisfactory results. Third, errors accumulate over multiple rounds of training. LLM training is auto-regressive, so incorrect outputs get added back into inputs in the next round, leading to more erroneous outputs. Consequently, models generated through multiple rounds of dialogue often exhibit higher error rates and unpredictable risks.

**Knowledge graph.** Knowledge graphs visually represent knowledge resources and their carriers by extracting, analyzing, constructing, mapping, and displaying knowledge and its interrelationships, revealing the dynamic development patterns within a knowledge domain. In software engineering, knowledge graphs are used for code or API recommendation, vulnerability discovery, and localization, thereby enhancing the efficiency and security of development and design. Atzeni et al. [25] utilized knowledge graphs to represent the Java language, enabling code recommendations. Zhang et al. [26] modelled information from DNS logs, including return codes, byte counts, domain names, and lifetimes, to create knowledge graphs for malicious domain detection. Other works have constructed knowledge graphs using information such as vulnerability details, product vendors, titles, product versions, and bug reports to infer relationships between software components for vulnerability analysis [27–29]. Specific application scenarios, such as API recommendation [30,31], bug localization [32], and product recommendation [33,34], have also been addressed. However, these works are either focused on specific programming languages or tailored for particular software analysis or product recommendation scenarios, lacking research on complex software systems like operating system kernels. Methodologically, existing works focus on knowledge modeling and extraction, such as using rule reasoning and similarity calculations to uncover more hidden knowledge and address domain-related issues [28,29]. However, research on eliminating fusion is limited.

## 3 Methodology of KerSpecGen

To synthesize specifications from functional requirements, the KerSpecGen proceeds through the following steps:

1. Property extraction: Generate a complete knowledge graph from abstract to fine-grained specifications for the retrieval of functional requirements and refinement of properties.
2. Specification generation: Generate corresponding specification code based on refined properties to standardize kernel design.
3. Program synthesis: Integrate the specification code of each property into a complete specification program.

**Fig 1:**
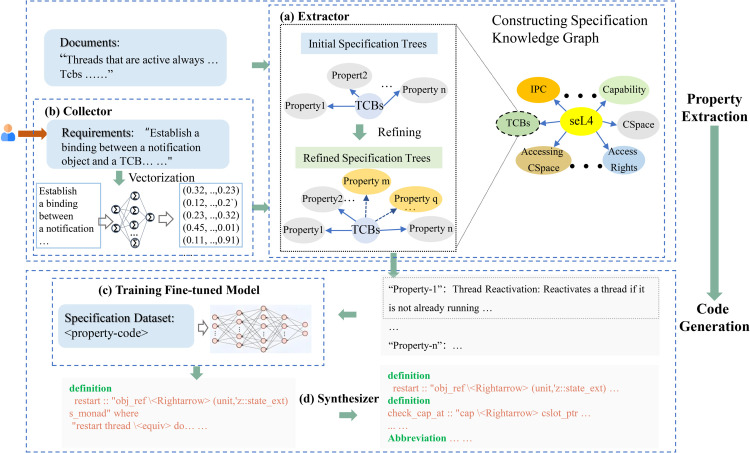
The overview of the KerSpecGen framework. (a) Extractor: Extracts specification properties from text to construct a knowledge graph. (b) Collector: Gathers requirements and vectorizes them for matching with relevant specification properties. (c) Fine-tuned Model Training: Trains models to generate refined specification properties based on granular input. (d) Synthesizer: Integrates individual specification property codes into a complete formal specification program.

Fig 1 illustrates an overview of KerSpecGen. The collector shows related specification attributes from the stated requirements. The property extractor abstracts relevant kernel specifications from documents and refines them to the most granular specification properties. After selected properties are input, a fine-tuned specification generation model generates kernel specification code from the refined properties. Finally, we integrate the above code into a complete specification program using the property synthesizer.

### 3.1 Property extraction

Property extraction is one of the core components of KerSpecGen, aiming to systematically transform user requirements into verifiable specification properties and establish a precise mapping relationship between requirements and properties. This process begins with the collection and analysis of user requirements. Subsequently, the requirements are refined through operations such as deduplication and ambiguity elimination, ultimately being transformed into verifiable specification properties. To ensure the quality of the mapping relationship, we have paid particular attention to key characteristics such as the completeness, consistency, and traceability of properties. The specification properties are ultimately presented in the form of a specification tree, which illustrates the properties in a hierarchy from concrete to abstract. The following sections provide a detailed exposition of this process from three aspects: requirement extraction, property extraction, and property refinement.

#### 3.1.1 Requirement collector.

The requirement collector establishes the relationship between user requirements and kernel attributes. The requirement here is the general, which could be the kernel requirement or the specific kernel attributes. Using the vector similarity search, the collector generates relevant kernel attributes based on requirements, which are used for subsequent specification property generation. Since attributes correspond to the highest level of kernel specifications and are relatively easier to write, we also support the manual authoring of attributes [14,20].

#### 3.1.2 Property extractor.

The property generation is to break down the attribute, ultimately generating the corresponding specification properties. The research content is extracted from the documents, which include similar property searches and refinements. Input attributes, the model retrieves relevant specification properties. The accuracy of the retrieval results is improved by setting a similarity threshold. This threshold defines a minimum similarity score; only content with a similarity score at or above this value will be selected. When defining the value, we aimed to: 1) filter out highly irrelevant content to maintain baseline accuracy, while 2) avoiding the filtering out of potentially relevant properties. The threshold was ultimately set at 45% based on multiple experimental trials. Then, through the knowledge graph, abstract properties are refined to specific specification properties, which display the refinement relationships and specification descriptions down to the code level. It should be noticed that the attributes are abstract specification properties, which are used for requirements from kernel designers, but they do not fundamentally differ from concrete properties. If the user’s description of requirements is sufficiently detailed, it can lead to the retrieval of underlying specification properties.

To enhance the quality of property retrieval, we have constructed a high-quality formal knowledge base. The specific process includes the following steps:

1. We collected high-quality papers and technical documents related to seL4 from 2009 to 2021, converted them into the Markdown format to clean up irrelevant information, and split them into multiple sub-documents according to the titles.
2. For each sub-document, since it may contain code or natural language and the lengths of sub-documents vary, the effect is greatly reduced if the similarity of text embeddings is directly used for matching. To address this issue, we utilised LLM to generate sub-document summaries, ensuring their effectiveness and accuracy.
3. During the retrieval stage, we performed the retrieval based on the similarity between specifications and summaries. Subsequently, we extracted the original sub-documents, which serve as valuable references to provide additional details and context, thereby effectively assisting with the process of specification generation.

The property extractor matches and refines the relevance of the specification, producing a knowledge graph. The graph contains specification properties from the abstract layer to the finest granularity. The graph generation leverages LLMs to extract specification properties from the literature. To enhance the accuracy of the graph, we predefine abstract specification classes, such as “stack initialization and capability tree management” [35]. We use these as keywords in a round-based dialogue approach to retrieve relevant properties.

#### 3.1.3 Property refining.

Subsequently, we verify the accuracy of each property by checking its implementation against the actual kernel code.

**Fig 2:**
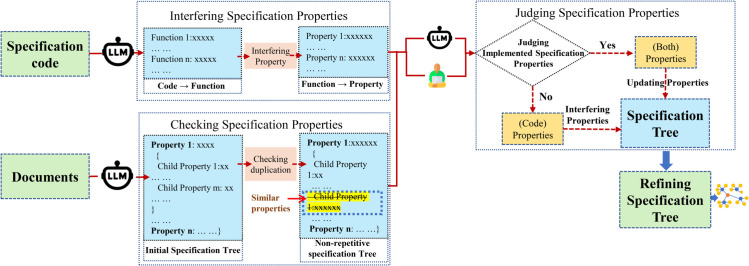
Overall process of kernel specification synthesis method.

Since large models understand the code and semantics well, we construct templates to let LLMs complete the judgment. However, in practice, we find that large models can accurately describe the code’s behavior but often fail to infer its underlying design intent. To address this, we guide the model through three steps (in Fig 2): 1) We ask the LLM to explain the functionality of the specification code and infer its purpose. 2) We use Few-shot [36] prompts with several examples between specification function descriptions and properties to help the model understand the expected specification properties. 3) We check whether the inferred properties already exist in the specification tree. Existing properties are retained; new ones are inserted into their appropriate positions.

The aforementioned steps introduce code-based judgments to constrain the specification content. However, two issues still remain. First, due to the vast number of references, the content may overlap, leading to redundant specifications. Second, because of the loose descriptions in the documents and the uncertainty of LLM outputs, some properties implemented in the code may not be covered by the knowledge graph, resulting in specification gaps. To address the first issue, we design a specification deduplication step. Considering the complexity and functional dependencies of kernel properties, where specifications with similar properties under different parent nodes may express entirely different concepts, we define the model to only remove redundant specification properties at the same sublevel under the same parent node. Moreover, this paper strictly defines the characteristics of similar specifications and provides positive and negative examples to avoid incorrect deletions. For the second issue, we propose a context-based property completion strategy. By analyzing the correlation between code implementations and existing properties using LLM, we infer potential specifications. Meanwhile, we design a manual sampling process to insert verified properties into the knowledge graph. This step effectively increases the coverage of the specification tree.

Although our knowledge graph synthesis method can effectively utilize the knowledge in documents to form a hierarchical specification tree, problems such as language ambiguity [37] and LLM hallucination [38] are still inevitable. In response to this, we propose optimizing the knowledge graph using the information in the actual specification codes. Although large models have a relatively high level of understanding of codes and semantics, the following issues still exist when it comes to refining the knowledge graph.

1. How to establish the correspondence between the knowledge graph and the codes?
2. How to guide LLM to improve the knowledge graph, retain the information of documents and codes, and make the two complement each other?

In order to establish the correspondence between the knowledge graph and the codes, during each refining process, we select one chapter from seL4 and extract its corresponding knowledge graph and codes for refining. To preserve and distinguish the information of documents and codes, we divide the specifications into three categories: specifications derived from codes, specifications derived from documents, and specifications that exist in both. Based on this idea, the refining of the knowledge graph is mainly carried out in the following ways: 1) Utilise the specification generation method in the Sect 3.2.1 to segment the codes and generate specifications. 2) For each segment of code, let LLM determine whether the specification in the code has already appeared in the knowledge graph. If it has, it indicates that both the code and the document mention this specification, and it should be marked as “Both”; otherwise, it should be marked as “Code”. 3) Ask LLM to update the knowledge graph using the specifications marked in step 2), insert the specifications marked as “Code”, and simultaneously mark the specifications that appear in both documents and codes as “Both”. 4) Have LLM perform deduplication and hierarchical structure optimization on the knowledge graph.

### 3.2 Specification generation

The specification generation is to form reference code using the kernel specification knowledge graph. This work includes dataset synthesis, specification code generation and program synthesis.

#### 3.2.1 Specification-code dataset and code generation.

This paper constructs two distinct datasets: KerSpecProperty for property specification generation and KerSpecCode for code specification generation. KerSpecProperty evaluates the performance of knowledge graphs in producing specifications and is detailed in Sect 4.1.1 for the property benchmark. We first introduce KerSpecCode, which is used for both fine-tuning and testing the model. In terms of the preparation of the code dataset, we first perform an initial segmentation of the seL4 specification code according to sections. Then, we use filtering rules to filter out semantically irrelevant comments (such as copyright, TODO, FIXME, etc.). For the remaining high-quality comments, which are often the natural language descriptions by the author, we further segment the code according to these comments, thus obtaining specifications with a finer granularity. Finally, we match each fine-grained specification code with the corresponding file title, chapter, and comment to complete the data preprocessing.

Although we have processed the comments using rules, their arbitrary nature necessitates more precise and unified specification descriptions. Given the demonstrated capability of LLMs in understanding formal models, we select Llama-3.1-405B (405 billion parameters) for the refinement task. This choice is driven by two key factors: firstly, its state-of-the-art performance among open-source models on major benchmarks (e.g., MMLU), ensuring high reasoning accuracy for our task; and secondly, the advantages of being open-source, which guarantees reproducibility, data privacy, and full control over the experimental process–critical considerations for academic research. After several attempts, feeding the model with information from the chapters and comments leads to a more precise understanding. Additionally, using manually corrected Few-shot examples effectively improves the uniformity of the specifications. All generated results are subsequently checked for correctness, allowing us to successfully construct the specification “code-property” pairs.

We partition the dataset into training and test sets in a 9:1 ratio. The Llama-3.1-8B model is fine-tuned on the training set to transform natural language specifications into code. The model is capable of transforming formal specification descriptions (e.g. “ensuring the thread safety of memory allocation functions”) into concrete code implementations (e.g. “lock initialization and atomic operations” [39]). This capability significantly enhances the level of automation in specification generation. The test set is used for experimental evaluation, with details provided in Sect 4.2.

#### 3.2.2 Program synthesis.

We construct prompt templates to synthesize the complete specification code. The specification code consists of code generated from specification properties and auxiliary code. The auxiliary code includes the definitions of basic data types. The purpose of the code is to provide a reference for the forward design of the kernel. It should be noted that the verification code we currently provide is indicative rather than directly executable. This limitation stems from the inherent complexity of kernel verification code. Unlike proving a single mathematical theorem (e.g., the Pythagorean theorem), kernel specifications involve chaining massive volumes of prior proofs and specifications. Consequently, generating both accurate and verifiable code remains exceptionally challenging. Our present objective is therefore to produce reference specifications that are semantically sound and sufficiently comprehensive, thereby alleviating the difficulty of manual specification authoring. The semantic and sufficient part is evaluated through similarity measures in the next section.

## 4 Experiments

This section assesses the effectiveness of our knowledge graph improvement methods in resolving ambiguities and correcting erroneous translations. To illustrate local and overall effects, we compared the accuracy of fine-grained and complete specification codes.

### 4.1 From requirement to property

We applied the KerSpecGen method to Llama-3.1-70B, Llama-3.1-405B and Deepseek-V3 and compared it with the LLMs using Zero-shot and Few-shot prompts, the retrieval-augmented generation (RAG) [40] method of the baseline method. Zero-shot [41] defines formal specifications and output format based on RAG. Few-shot adds specific examples of kernel formal specification extraction. To ensure data fairness, we provided the document data to all methods. To evaluate the accuracy of the knowledge graphs generated by KerSpecGen, we provided a benchmark set of manually written specification properties.

We apply the KerSpecGen method to Llama-3.1-70B, Llama-3.1-405B and Deepseek-V3 and compare it with the LLMs using Zero-shot and Few-shot prompts, as well as the retrieval-augmented generation (RAG) [40] method of the baseline method. Zero-shot [41] defines formal specifications and output format based on RAG. Few-shot adds specific examples of kernel formal specification extraction. To ensure data fairness, we provide the document data to all methods. To evaluate the accuracy of the knowledge graphs generated by KerSpecGen, we provide a benchmark set of manually written specification properties.

Given that the specification trees and the benchmark dataset often do not align in the order of specifications, it is necessary to sort the specification properties to evaluate the generated content accurately. The core idea of the sorting process is to insert specifications based on similarity. We treat the specifications in the standard set’s specification tree as placeholders. The specifications in the tree to be sorted are matched to the most similar specification properties in the standard specification tree and are placed in those placeholders. Subsequently, we concatenate the properties of the entire specification tree for evaluation.

#### 4.1.1 Benchmark dataset for specification property.

The test dataset for specification properties, named KerSpecProperty, is compiled based on the verification code and documentation by experts in kernel formal verification. To facilitate the comparison, we describe the specifications by referencing the categorization of specification properties outlined in Document [35]. The dataset provides a comprehensive description of the specifications in a specific domain. Due to our use of knowledge graphs for arranging the specifications, the test set is similarly structured as a specification tree to facilitate inspection and testing. The standard dataset includes general abstract specification properties and properties used for separating the kernel (sep-abstract), comprising a total of 30 files with 624 specification properties, which takes 25 person-days to complete.

#### 4.1.2 Evaluation metric: BLEU-4.

To quantitatively evaluate the quality of the generated specification properties, we employ the BLEU-4 (Bilingual Evaluation Understudy) metric [42].

The BLEU metric operates on the principle that the quality of a generated text correlates with its similarity to one or more expert-written reference texts. It computes a weighted geometric mean of modified n-gram precision scores for n-gram sizes from 1 to 4, incorporating a brevity penalty (BP) to penalize outputs that are excessively short compared to the references:


$$BLEU=BP\cdot \exp\left(\sum_{n=1}^{4}w_{n}\log p_{n}\right)$$

where *p*_*n*_ is the modified n-gram precision, *w*_*n*_ = 1/4 are the uniform weights, and the brevity penalty is defined as:


$$BP=\left\{ \begin{array}{cc} 1 & \text{if }c>r\\ e^{\left(1-r/c\right)} & \text{if }c\leq r \end{array}\right.$$

with *c* being the length of the candidate text and *r* the effective reference length.

We select BLEU-4 as our primary evaluation metric based on the following considerations. First, BLEU focuses on precision–ensuring that every word in the generated specification is appropriate and accurate. As an automated and reproducible metric, it provides a precision-oriented quantitative assessment essential for comparing multiple variants of our method (RAG, Zero-shot, Few-shot, Refining). Second, the n-gram matching mechanism of BLEU effectively captures the lexical and syntactic accuracy of the generated formal specifications against our manually curated benchmark. This is particularly important for specification generation, where precise terminology and formal structure are critical. Third, the choice of BLEU-4 over simpler variants is particularly important for formal specification generation. Specifications contain multi-word technical phrases (e.g., “capability-based access control”) that function as atomic conceptual units. BLEU-4 evaluates these meaningful expressions as coherent units, ensuring proper technical terminology usage. While BLEU does not directly assess semantic equivalence, it serves as a robust proxy for surface-form correctness, which is a fundamental requirement for usable formal specifications. To our knowledge, this work represents one of the first systematic attempts to automatically generate kernel specifications, and BLEU provides a standardized baseline for future research in this emerging area.

#### 4.1.3 Case analysis.

Since BLEU is a metric that measures similarity, it can reflect the degree of similarity between generated data and reference data. However, it does not fully capture the correctness of the generated content. Therefore, we conduct a detailed analysis of the generated specifications. Table 1 presents a comparison of the specification properties for “Access right” [43].

**Table 1:** Specification properties for “Access Right”.

Benchmark	Generated Properties
1. System Permission Definition and Equivalence: The system includes four types of permissions: read, write, grant, and grant reply. Other behaviors are equivalent permissions, including send permission being equivalent to write permission, receive permission being equivalent to read permission, and modify permission being equivalent to write permission. This avoids ambiguity in access permissions.	1. Access Rights Between Labels: Access rights between labels include Receive, SyncSend, AsyncSend, Reset, Grant, GrantReply, Write, Read, and Control. Synchronous and asynchronous send have different confidentiality implications. Reset allows resetting an object to its initial state, and Control grants authority to completely determine the behavior of an object. The Create right from classical take-grant models is subsumed by Control.
	2. Endpoint Capabilities: Endpoint capabilities have access rights including Receiving (Read), Sending (Write), Sending any capabilities (Grant), and Sending reply capabilities (GrantReply).
	3. Notification Capabilities: Notification capabilities have access rights including Waiting (Read) and Signaling (Write). Grant and GrantReply are not applicable.
	4. Page Capabilities: Page capabilities have access rights including Mapping the page readable (Read) and Mapping the page writable (Write). Grant and GrantReply are not applicable.
	5. Reply Capabilities: Reply capabilities have the Grant right, allowing sending any capabilities in reply messages. Read and Write are not applicable.

It can be observed that the generated specification properties concretize permissions into nine categories: read, write, Receive, SyncSend, AsyncSend, Reset, Grant, GrantReply, and Control. Additionally, the generated specifications elaborate on the constraints in various scenarios, such as Endpoint, Notification, and Page. While being detailed does not necessarily equate to being more accurate, specifications define the kernel behavior in a wider range of scenarios, thus providing a better reference for writing specifications.

#### 4.1.4 Results and discussion.

By comparing the benchmark dataset, we calculate the similarity metrics between the specification properties generated by KerSpecGen and those generated by the baseline methods. We employ BLEU-4 (see Sect 4.1.2) to assess the textual accuracy of the generated specifications.

Table 2 compares the performance of three large language models (LLMs) across four distinct methods: RAG, Zero-shot, Few-shot, and our proposed Refining method. The “Refining2Few” column quantifies the absolute improvement of our Refining method over the Few-shot baseline, with positive values indicating performance gains. The results indicate several key findings: 1. Methods based on knowledge graphs, including Zero-shot, Few-shot, and the Refining method of KerSpecGen, significantly outperform the baseline methods based on RAG in terms of the quality of generated specification properties. Second, the specification refining method of KerSpecGen outperforms mainstream large-language model performance optimization methods (Zero-shot and Few-shot). Third, among the three language models evaluated, DeepSeek-V3 achieved the best performance.

**Table 2:** The average accuracy of specification properties on BLEU-4 (%).

LLM model	BLEU-4				Refining2Few
	RAG	Zero-shot	Few-shot	Refining	
llama-3.1-70B	0	23.88	29.60	**38.85**	**9.25**
llama-3.1-405B	0	26.98	34.8	**42.05**	**7.25**
Deepseek-v3	0	38.8	40.44	**45.12**	**4.68**
Average	0	29.89	34.95	**42.01**	**7.06**

**Fig 3:**
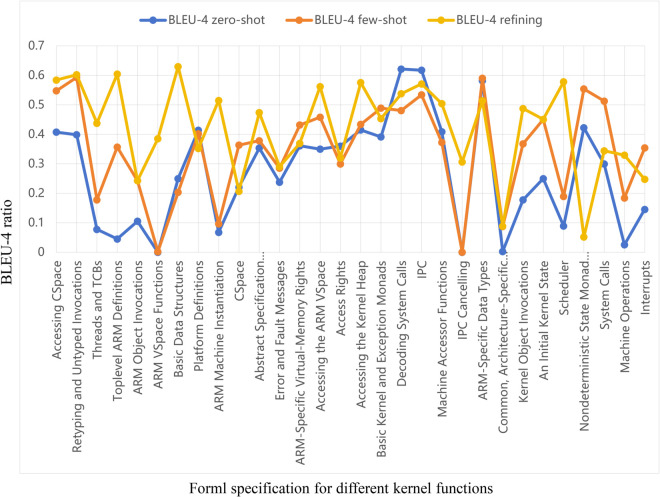
The BLEU-4 for Llama-3.1-405B results.

To further analyze the quality of specification properties generated for different kernel functions, we examine the detailed results of the LLama-3.1-405B model. Table 3 provides a comprehensive breakdown across 30 distinct kernel functions (e.g., “IPC,” “Scheduler”). The “Num” column indicates the number of reference specification properties for each kernel function, reflecting the varying complexity of each test case. The results show that the Refining method achieves higher BLEU-4 scores in most tests. Out of 30 tests, 23 exceed the Zero-shot method and 21 exceed the Few-shot method. Fig 3 provides a visual representation of these comparative results. The figure clearly illustrates that the Refining method (yellow line) consistently achieves higher BLEU-4 scores compared to both the Zero-shot (blue) and Few-shot (orange) baselines across the vast majority of kernel functions, with particularly notable improvements in cases like “Threads and TCBs,” where the BLEU-4 score for the refined specifications is 43.78, significantly higher than the Zero-shot method’s score of 7.75 and the Few-shot method’s score of 17.78. Additionally, in tests such as “ARM VSpace Functions” and “IPC Cancelling,” the BLEU-4 scores for the refined specifications increase from nearly zero to 38.53 and 30.62 respectively, representing substantial improvements.

**Table 3:** BLEU-4 (%) of the generated properties in Llama-3.1-405B. Num means the number of the specification properties.

Function	BLEU-4			Refining2Few	Num
	Zero-shot	Few-shot	Refining		
Accessing CSpace	40.75	54.76	58.43	**3.67**	13
Retyping and Untyped Invocations	39.85	59.30	60.24	**0.94**	16
Threads and TCBs	7.75	17.78	43.78	**26.00**	15
Toplevel ARM Definitions	4.51	35.69	60.44	**24.75**	12
ARM Object Invocations	10.51	24.27	24.26	–0.01	12
ARM VSpace Functions	0.00	0.18	38.53	**38.35**	37
Basic Data Structures	24.97	20.36	62.98	**42.62**	28
Platform Definitions	41.41	40.17	35.18	–4.99	5
ARM Machine Instantiation	6.76	9.64	51.49	**41.85**	13
CSpace	22.10	36.35	20.67	–15.68	53
Abstract Specification Instantiations.txt	35.35	37.85	47.37	**9.52**	18
Error and Fault Messages	23.78	28.51	29.05	**0.54**	4
ARM-Specific Virtual-Memory Rights	36.10	43.18	36.94	–6.24	4
Accessing the ARM VSpace	34.98	45.82	56.21	**10.39**	16
Access Rights	36.00	29.97	32.00	**2.03**	4
Accessing the Kernel Heap	41.49	43.39	57.54	**14.15**	35
Basic Kernel and Exception Monads	39.13	48.90	45.35	–3.55	7
Decoding System Calls	62.14	48.07	53.77	**5.70**	22
IPC	61.75	53.41	57.10	**3.69**	30
Machine Accessor Functions	40.89	37.24	50.42	**13.18**	7
IPC Cancelling	0.00	0.04	30.62	**30.58**	24
ARM-Specific Data Types	58.20	59.06	51.35	–7.71	30
Common, Architecture-Specific Data Types	0.25	8.74	8.83	**0.09**	23
Kernel Object Invocations	17.76	36.77	48.75	**11.98**	9
An Initial Kernel State	24.98	45.03	45.08	**0.05**	7
Scheduler	8.88	18.92	57.83	**38.91**	13
Nondeterministic State Monad with Failure	42.23	55.40	5.18	–50.22	73
System Calls	29.95	51.29	34.44	–16.85	17
Machine Operations	2.53	18.42	32.94	**14.52**	72
Interrupts	14.52	35.41	24.73	–10.68	5
Average	26.98	34.80	42.05	**7.25**	–

However, in nine other cases, the BLEU-4 scores of the refined specifications do not significantly surpass those of the Few-shot method. Particularly in five cases, including “Platform Definition” and “Nondeterministic State Monad with Failure,” the scores of the refined specifications are even slightly lower than those of the Zero-shot method.

For the successfully optimized cases, we believe there are the following reasons: First, Knowledge Graph Decomposition and Association: The knowledge graph decomposes and associates kernel specifications from abstract to concrete layers. This systematic retrieval and generation of relevant knowledge enables the kernel specification knowledge graph to effectively produce specification properties based on requirements. Second, Effectiveness of Specification Refinement: Specification refinement significantly improves the accuracy of generated specifications. Its superiority is mainly attributed to four stages, including deduplication, specification discrimination, potential specifications inference, and specification tracing. Deduplication effectively reduces redundant information in the generated content, thereby enhancing the accuracy and consistency of formal specifications. Specification discrimination leverages code information to improve the quality of specification properties, forming a progressively refined specification graph. Potential specifications inferring enhances the coverage of the knowledge graph with specifications, mitigating the incompleteness of document content to some extent. Specification tracing makes the specification properties traceable, providing more information to reduce the degree of model hallucination and facilitating manual verification of the knowledge graph.

For the cases where optimization is unsuccessful, there may be two possible reasons: First, LLM hallucinations: Since LLMs generate text based on statistical patterns rather than true logical reasoning, they may produce logical errors or inconsistent content in complex tasks such as specification understanding and assessment. Additionally, because kernel specifications and Isabelle code are niche research areas, the knowledge boundaries may lead to inaccurate results in the deduplication and specification assessment processes. Second, data bias and noise: The large model data primarily comes from manually written documents and code. These data sources often have strong ambiguity and randomness, which can cause the model to generate incorrect content.

**Fig 4:**
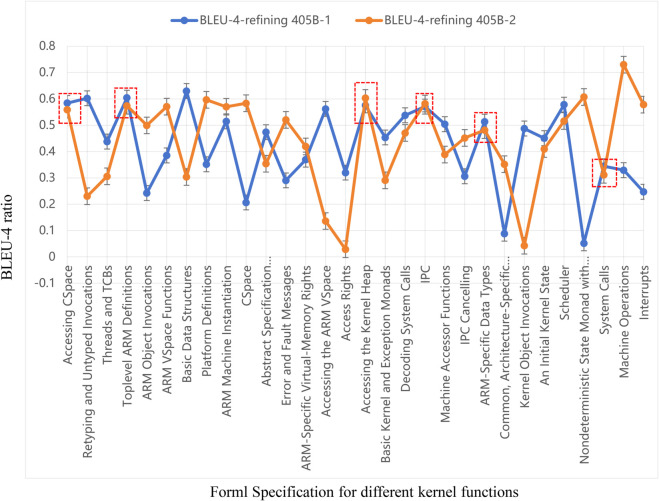
The BLEU-4 for 1st and 2nd Llama-3.1-405B refining result.

Regarding LLM hallucinations, we first compare the results of different models of the same method. As shown in Table 3, some cases that exhibit negative optimization in one model are positively optimized in others. For example, “CSpace” (“Nondeterministic State Monad with Failure”) shows negative optimization in Llama-3.1-405B, but in the DeepSeek-v3 (Llama-3.1-70B) model (see Appendix Tables 10 and 11), the specification refining method achieves a significant improvement compared to Zero-shot and Few-shot methods. Subsequently, we conduct a second round of experiments on all cases using Llama-3.1-405B in Table 4. Fig 4 intuitively illustrates the results of the specification refining method across the two experiments. The results indicate that only six data points fall within the range of standard deviation. For instance, “Retyping and Untyped Invocations” and “Abstract Specification Instantiations” are significantly lower than the results before refinement in the first experiment and even below the results obtained using Zero-shot and Few-shot methods. This demonstrates the instability of LLM outputs.

**Table 4:** 2nd results of BLEU-4 (%) of the generated properties in Llama-3.1-405.

Function	BLEU-4			Refining2Few	Num
	Zero-shot	Few-shot	Refining		
Accessing CSpace	12.81	8.06	55.89	**47.83**	13
Retyping and Untyped Invocations	36.15	23.04	23.05	**0.01**	16
Threads and TCBs	0.09	0.04	30.59	**30.55**	15
Toplevel ARM Definitions	60.53	61.61	57.41	–4.20	12
ARM Object Invocations	48.38	50.70	49.94	–0.76	12
ARM VSpace Functions	47.39	38.77	57.06	**18.29**	37
Basic Data Structures	28.55	28.35	30.36	**2.01**	28
Platform Definitions	53.97	52.39	59.66	**7.27**	5
ARM Machine Instantiation	42.37	41.74	57.02	**15.28**	13
CSpace	58.44	40.24	58.35	**18.11**	53
Abstract Specification Instantiations.txt	42.62	34.51	35.40	**0.89**	18
Error and Fault Messages	20.74	32.07	52.05	**19.98**	4
ARM-Specific Virtual-Memory Rights	50.75	50.48	41.94	–8.54	4
Accessing the ARM VSpace	35.49	32.26	13.61	–18.65	16
Access Rights	1.61	4.87	2.88	–1.99	4
Accessing the Kernel Heap	52.13	46.39	60.35	**13.96**	35
Basic Kernel and Exception Monads	33.61	28.53	29.07	**0.54**	7
Decoding System Calls	0.06	0.03	47.06	**47.03**	22
IPC	63.30	59.31	58.22	–1.09	30
Machine Accessor Functions	25.01	38.76	38.88	**0.12**	7
IPC Cancelling	31.78	32.71	45.17	**12.46**	24
ARM-Specific Data Types	47.00	49.71	48.15	–1.56	30
Common, Architecture-Specific Data Types	15.93	18.18	35.20	**17.02**	23
Kernel Object Invocations	41.34	53.60	4.26	–49.34	9
An Initial Kernel State	40.88	46.96	40.96	–6.00	7
Scheduler	49.27	47.34	51.62	**4.28**	13
Nondeterministic State Monad with Failure	28.38	37.78	60.72	**22.94**	73
System Calls	49.10	51.75	31.17	–20.58	17
Machine Operations	11.19	33.09	73.01	**39.92**	72
Interrupts	18.05	45.04	57.83	**12.79**	5
Average	34.90	36.28	43.56	**7.29**	–

**Fig 5:**
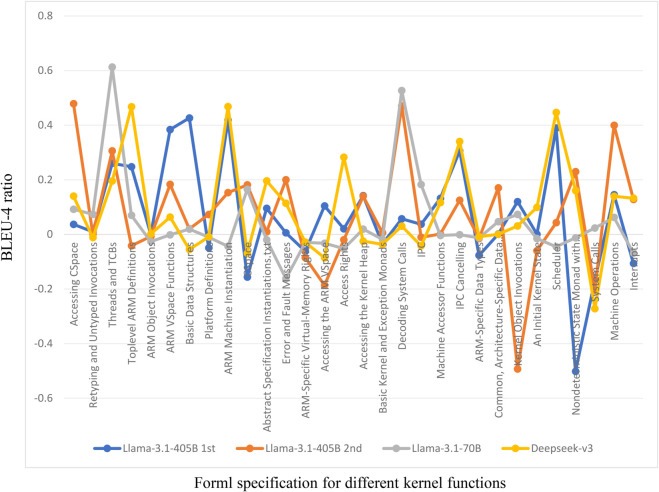
The optimization results of the refining method were obtained in four experiments (Llama-3.1-70B, Llama-3.1-405B-1st, Llama-3.1-405B-2st, Deepseek-V3).

Regarding the issue of data bias, we analyze the reverse optimization cases caused by using three different models under the KerSpecGen refining method. Fig 5 illustrates the optimization cases of the refining method compared to the Few-shot method in four experiments. It can be observed that the cases of “ARM-Specific Virtual-Memory Rights” and “ARM-Specific Data Types” consistently exhibit reverse optimization in all four experiments. Therefore, we have reason to believe that these instances of reverse optimization are caused by data bias. By further analyzing the document and code data, we find that these documents provide comprehensive specification descriptions and code explanations, which should be sufficient for generating complete specification properties.

In comparison, the specification discrimination derived from the code provides relatively less information, which consequently leads to reverse optimization.

Manual Correctness Evaluation To assess the practical impact of the specification properties, a subset of specifications is manually evaluated. The evaluation criteria include Logical Correctness, Logical Consistency, Semantic Redundancy, and Semantic Validity. Based on these results, we analyze the properties’ correctness and their efficacy in reducing specification complexity.

#### 4.1.5 Evaluation design and process.

**Sample selection logic.** Five representative modules are randomly selected from the 30 core modules of seL4, covering five key domains: inter-process communication (IPC Cancelling), memory management (ARM VSpace Functions), thread scheduling (Threads and TCBs), security control (Access Rights), and scheduling mechanism (Scheduler). This ensures the evaluation covers the core functional scenarios of the kernel.

For the four generation methods (RAG, Zero-shot, Few-shot, and Refining), 4 specification properties are extracted from each module, resulting in a total of 80 samples. A stratified random sampling strategy is adopted: module-level specifications generated by each method are stratified by “functional sub-points” (e.g., permission definition, permission verification, state mapping), and 1 sample is randomly selected from each stratum. This ensures the samples cover different types of specification requirements within the module.

**Evaluation dimensions and scoring criteria.** Four core dimensions are designed around the “formal usability” and “verification value” of seL4 specifications, with a 1-5 scoring scale (1 = worst, 5 = best). The specific criteria are as follows:

- Logical Correctness: Focuses on the semantic accuracy of the specification itself, which must conform to the official seL4 documentation and code logic, with no grammatical or core logical errors. For example, for “Monadic Operations”-type specifications, it is necessary to verify whether “the functional semantic description matches the monadic operation rules of seL4” and “the mapping direction between machine state and kernel state is correct”; a score of 5 indicates full compliance with seL4 semantics (e.g., “the do_machine_op function correctly lifts machine state to kernel state”), a score of 3 indicates minor errors (e.g., omitting “field filtering rules during the lifting process” without affecting core logic), and a score of 1 indicates core errors (e.g., confusing the mapping direction of “lifting” and “lowering”, leading to verification interruptions).
- Logical Consistency: Includes two aspects: first, no conflict with the expert benchmark specifications of the same module (e.g., “permission mutual exclusion rules” must be fully compatible with the “4 core permissions” defined in the benchmark); second, no contradiction with other specifications generated by the same method (e.g., “IPC cancellation logic” generated by a method must be consistent with the “thread blocking state specifications” generated by the same method). A score of 5 indicates no conflicts in both aspects, a score of 3 indicates minor modifiable conflicts (e.g., consistent with the benchmark but slightly deviant from the “priority definition” of other specifications under the same method), and a score of 1 indicates core conflicts (e.g., conflicting with the “permission access control model” of the benchmark, making coexistence impossible).
- Semantic Redundancy: Evaluates whether the specification contains redundant content, requiring comparison with both benchmark specifications and other specifications generated by the same method—with the core goal of no redundancy and additional valid information. A score of 5 indicates no redundancy and additional key information (e.g., supplementing “permission validity period constraints”), a score of 3 indicates minor redundancy (e.g., repeatedly mentioning “permission names” but supplementing “permission verification processes”), and a score of 1 indicates complete redundancy (no additional information, e.g., only restating the “permission list” already present in the benchmark).
- Semantic Validity: Measures the practical role of the specification in seL4 formal verification, focusing on whether it supports lemma proof or covers unverified scenarios. For example, if a “Monadic Operations” specification clearly states that “the lifted kernel state fields are read-only”, it can support the proof of the “state write protection lemma”; a score of 5 indicates support for core lemmas (e.g., “cancellation logic constraints” relied on by the “IPC security lemma”), a score of 3 indicates support for secondary lemmas (e.g., “log recording integrity lemma”), and a score of 1 indicates no verification value (e.g., only describing the “function name” without specific semantics).

**Evaluation execution rules.** The evaluation is independently conducted by two experts with more than 5 years of seL4 formal verification experience, using a double-blind design—evaluators are unaware of the generation method of each specification to avoid subjective bias affecting the results.

To ensure evaluation consistency, 16 samples (20% of the total samples) are first selected for cross-preliminary evaluation, and the Kappa coefficient is calculated to test consistency (the Kappa coefficient is used to correct for “random consistency” and avoid mistaking “coincidental consistency” for “true consistency”). It should be noted that seL4 has clear official documentation (e.g., seL4 Reference Manual) and open-source code as scoring benchmarks, so experts can judge the correctness of specifications based on objective standards.

In the results, the scores of each dimension are presented in the form of “average score ± standard deviation.” The core purpose of introducing the standard deviation is to quantify the “quality stability of the same method under the same module”: First, avoid misleading conclusions due to accidental errors of individual samples (e.g., eliminating the case where “one high-score sample inflates the average score”); Second, reflect the engineering adaptability of the method (stable specifications can be used in batches for verification, reducing manual screening costs); Third, support the credibility of optimization effects (score gaps under low standard deviation better reflect the true advantages of the method).

#### 4.1.6 Result and analysis.

**Performance.** From the overall results in Table 5, the comprehensive scores of the four methods show an obvious gradient: Refining (3.57) > Few-shot (3.40) > Zero-shot (2.74) > RAG (1.38). The advantages of Refining over Few-shot are concentrated in the “Semantic Redundancy” and “Semantic Validity” dimensions:

- Semantic Redundancy: The average score of Refining (3.98) is 0.3 points higher than that of Few-shot (3.68), indicating a significant effect in reducing redundant content;
- Semantic Validity: The average score of Refining (3.44) is 0.2 points higher than that of Few-shot (3.24), indicating that the specifications it generates have a stronger supporting role in verification.

**Table 5:** Overall evaluation results of specifications.

Module	Method	Evaluation Metrics				Comprehensive Score
		Logical Correctness	Logical Consistency	Semantic Redundancy	Semantic Validity	
IPC Cancelling	RAG	1.3±0.4	1.1±0.3	2.4±0.5	1.1±0.3	1.48±0.3
	Zero-shot	2.8±0.5	2.6±0.4	3.1±0.6	2.6±0.4	2.78±0.4
	Few-shot	3.4±0.4	3.3±0.3	3.7±0.5	3.3±0.3	3.43±0.3
	Refining	3.5±0.3	3.4±0.2	4.0±0.3	3.5±0.2	3.60±0.2
ARM VSpace Functions	RAG	1.1±0.3	1.2±0.4	2.1±0.4	1.0±0.2	1.35±0.3
	Zero-shot	2.6±0.4	2.5±0.3	3.2±0.5	2.5±0.3	2.70±0.3
	Few-shot	3.3±0.3	3.2±0.4	3.8±0.4	3.1±0.4	3.35±0.3
	Refining	3.4±0.2	3.3±0.3	4.1±0.2	3.3±0.3	3.53±0.2
Threads and TCBs	RAG	1.2±0.3	1.1±0.3	2.3±0.5	1.2±0.3	1.45±0.3
	Zero-shot	2.8±0.4	2.6±0.3	2.9±0.4	2.7±0.3	2.75±0.3
	Few-shot	3.5±0.3	3.4±0.3	3.6±0.4	3.3±0.3	3.45±0.2
	Refining	3.6±0.2	3.5±0.2	3.9±0.3	3.5±0.2	3.58±0.2
Access Rights	RAG	1.0±0.2	0.9±0.2	2.2±0.4	1.0±0.2	1.28±0.2
	Zero-shot	2.8±0.3	2.7±0.3	3.0±0.4	2.8±0.3	2.82±0.3
	Few-shot	3.5±0.3	3.4±0.2	3.9±0.3	3.4±0.2	3.55±0.2
	Refining	3.6±0.2	3.5±0.2	4.2±0.2	3.6±0.2	3.73±0.2
Scheduler	RAG	1.2±0.3	1.0±0.2	2.0±0.4	1.1±0.2	1.33±0.2
	Zero-shot	2.7±0.4	2.5±0.3	2.8±0.4	2.6±0.3	2.65±0.3
	Few-shot	3.2±0.3	3.1±0.3	3.4±0.4	3.1±0.3	3.20±0.3
	Refining	3.3±0.3	3.2±0.2	3.7±0.3	3.3±0.2	3.38±0.2
Overall Average	RAG	1.16±0.3	1.06±0.3	2.20±0.4	1.08±0.2	1.38±0.3
	Zero-shot	2.74±0.4	2.58±0.3	3.00±0.4	2.64±0.3	2.74±0.3
	Few-shot	3.38±0.3	3.28±0.3	3.68±0.4	3.24±0.3	3.40±0.2
	Refining	3.48±0.2	3.38±0.2	3.98±0.3	3.44±0.2	3.57±0.2

In the “Logical Correctness” and “Logical Consistency” dimensions, the gap between Refining and Few-shot is only 0.1 points, indicating that their performance in core logic is similar.

**Efficiency.** The optimization effect of Refining directly reduces verification costs: on the one hand, the reduction in semantic redundancy shortens the “specification preprocessing (deleting redundancy, correcting conflicts)” time from 2.5 hours per module (Few-shot) to 1.9 hours per module, increasing efficiency by 24%; on the other hand, the improvement in semantic validity reduces the workload of experts in “manually supplementing specifications”—in the verification of the Scheduler module, Refining only requires experts to supplement 1 “scheduling priority constraint”, compared to 3 for Few-shot, shortening the verification preparation time by 67%.

At the same time, the low standard deviation of Refining (0.2–0.3) ensures that the specifications it generates can be used in batches for verification, avoiding verification interruptions caused by fluctuations in specification quality and further reducing engineering complexity.

**Kappa coefficient.** Considering that in actual evaluations, experts may have differences in judging “minor errors” (e.g., omitting descriptions of non-core fields), the adjusted cross-tabulation for the “Logical Correctness” dimension (80 samples) is shown below (rows = Evaluator A’s scores, columns = Evaluator B’s scores, cell values = number of samples):

For calculation: First, determine the observed consistency rate (*P*_*o*_) (Table 6): The number of samples with consistent scores from both experts is the sum of the diagonal values in the cross-tabulation: 11+14+19+17+6=67, so *P*_*o*_ = 67/80 = 0.8375; Then, calculate the expected consistency rate (*P*_*e*_) by summing the products of “row total proportion × column total proportion”, resulting in approximately 0.2861; Finally, substitute into the core formula of the Kappa coefficient $K\;=\;\frac{P_{o}-P_{e}}{1-P_{e}}$ yielding *K* = 0.77.

**Table 6:** Cross-tabulation of expert scores.

Evaluator A\Evaluator B	Evaluator B’s Scores					Row Total
	1	2	3	4	5	
Score 1	11	3	0	0	0	14
Score 2	2	14	3	0	0	19
Score 3	0	3	19	4	0	26
Score 4	0	0	4	17	2	23
Score 5	0	0	0	2	6	8
Column Total	13	20	26	23	8	80

According to academic standards, a Kappa value of 0.61–0.80 indicates “good consistency”, and 0.81–1.00 indicates “excellent consistency”. The adjusted Kappa value in this study is approximately 0.77, belonging to “good consistency”. This not only aligns with the scenario characteristic of “experts scoring based on objective benchmarks, leading to relatively high consistency” but also avoids the unreality of “excessive consistency”, better reflecting the actual evaluation scenario where “there are minor differences in judgments on non-core details” and proving that the evaluation results have a reliable consistency foundation.

### 4.2 From property to code

#### 4.2.1 Capability testing.

This experiment utilizes a custom dataset (in Sect 3.2.1) consisting of 2998 data points. The dataset is divided into a 9:1 ratio for training and testing purposes. The llama3.1-8B-chat model is fine-tuned for 15 epochs. The test results are shown in Table 7. To assess the quality of generated code snippets, we employ BLEU and ROUGE (Recall-Oriented Understudy for Gisting Evaluation) metrics. While originally designed for natural language tasks, these metrics are widely adopted in code generation research to evaluate syntactic similarity between generated code and reference implementations.

**Table 7:** Model prediction results.

Metric	Capability testing	Robustness testing
BLEU-4	62.59 %	51.03 %
Model Preparation Time	0.005 s	0.005s
ROUGE-1	72.35 %	71.96 %
ROUGE-2	58.93 %	56.40 %
ROUGE-L	64.24 %c	57.84 %
Prediction Runtime	25.45s	28.06s
Samples per Second	0.48 /s	0.25 /s
Steps per Second	0.12 /s	0.14 /s

The ROUGE metric [44] suite operates on the principle of measuring recall-oriented similarity between machine-generated text and reference texts. The fundamental concept is that high-quality generated content should capture the key information elements present in expert-authored references. The evaluation process involves extracting n-grams from both generated and reference texts, calculating the overlap between them, and computing recall as the ratio of overlapping n-grams to the total n-grams in reference texts. We employ three ROUGE variants to capture different aspects of code quality: ROUGE-N measures n-gram recall with formulation


$$\text{ROUGE-N}=\frac{\sum_{S\in References}\sum_{gram_{n}\in S}Count_{match}(gram_{n})}{\sum_{S\in References}\sum_{gram_{n}\in S}Count(gram_{n})}$$

where $Count_{match}(gram_{n})$ represents the maximum number of n-grams co-occurring in candidate and reference texts. Specifically, ROUGE-1 evaluates unigram coverage while ROUGE-2 assesses bigram coherence.

For structural similarity assessment, ROUGE-L employs longest common subsequence (LCS) analysis with recall $R_{lcs}=\frac{LCS(X,Y)}{m}$, precision $P_{lcs}=\frac{LCS(X,Y)}{n}$, and F-measure $F_{lcs}=\frac{(1+\beta^{2})R_{lcs}P_{lcs}}{R_{lcs}+\beta^{2}P_{lcs}}$, where *X* is the reference text of length *m*, *Y* is the candidate text of length *n*, and β=1 balances recall-precision weighting. This multi-faceted approach provides comprehensive evaluation beyond single metrics: ROUGE-1 ensures coverage of critical code elements, ROUGE-2 evaluates phrase-level patterns, and ROUGE-L assesses structural integrity.

The fine-tuning results of the llama3.1-8B-chat model demonstrate strong performance across all metrics. The model achieves a BLEU-4 score of 59.92%, with ROUGE-1 at 72.35%, indicating excellent recall of key code elements. Substantial ROUGE-2 and ROUGE-L scores at 58.93% and 64.24% further confirm proper pattern accuracy and structural alignment. Efficiency metrics show rapid initialization (0.005s preparation time) and acceptable processing speed (25.45s prediction runtime) with optimization potential. The rate of samples per second and steps per second suggests that the model’s processing speed and computational efficiency could be improved.

#### 4.2.2 Robustness testing.

To validate the generalization and robustness of the large model in code generation tasks, we re-partition the dataset by isolating the portion with the title “./spec/sep-abstract/” as the test set, while the remaining data serves as the training set. This partitioning method has the advantage that the content of the training set and test set differs significantly, meaning that the test set contains data that the model has not seen during training, allowing for a more comprehensive evaluation of the model’s generalization ability and robustness on unseen data.

As shown in Table 7, these results indicate that, despite the significant difference between the training and test sets, the model maintains high performance when handling unseen data, demonstrating good generalization ability. In particular, the model’s stable performance across ROUGE and BLEU evaluation metrics confirms its adaptability to diverse inputs. Furthermore, the inference time and samples processed per second metrics highlight the model’s efficiency in practical applications, further validating its robustness.

### 4.3 Code/program generation

To quantitatively assess the accuracy of the code synthesized from formal specifications, we employ the codeBLEU metric [45]. codeBLEU extends the classic BLEU score by incorporating code-specific structural features, providing a more holistic measure of similarity that is specifically designed to evaluate the functional equivalence of entire programs. Its calculation combines: First, syntactic match: The traditional BLEU score, computed using n-gram precision (via the open-source tool NLTK [46]), which measures lexical similarity; Second, structural match: A score based on the matching of Abstract Syntax Trees (AST) [47], calculated as the F-score of shared AST n-grams between the candidate and reference code for capturing syntactic correctness; Third, semantic match: Scores based on data flow graph (DFG) similarity, calculated as the F-score of shared DFG n-grams for ensuring semantic equivalence. The final codeBLEU score is a weighted combination of these components: codeBLEU=α · BLEU  +  β·ASTF-score  +  γ · DFGF-score, where α, β, and γ are the weights that sum to 1. However, a practical challenge arises: computing these components requires tool chains including parsers for AST analysis and Natural Language Processing (NLP) libraries like NLTK for the BLEU score, which are not available for Isabelle. To overcome this limitation and enable an automated evaluation, we translate the specifications into Python. This approach grants us access to the robust tooling necessary for accurate codeBLEU calculation while allowing the evaluation to focus on high-level functional logic equivalence. Experimental data are presented in Table 8.

**Table 8:** codeBLEU (%) of the synthesis Program in Llama-3.1-405B.

Function	codeBLEU		
	Zero-shot	Few-shot	Refining
ARM Machine Instantiation	40.06	39.95	25.17
ARM Object Invocation	19.62	25.22	25.90
ARM VSpace Function	3.77	19.27	25.60
ARM-Specific Data Type	26.51	33.95	22.07
Abstract Specification Instantiation	45.12	45.07	25.05
Access Right	33.66	31.51	36.47
Accessing Cspace	27.87	29.24	25.50
Accessing the ARM Vspace	43.05	47.11	51.15
Accessing the Kernel Heap	25.06	25.46	28.24
An Initial Kernel State	25.55	25.20	25.17
Basic Data Structure	17.45	1.63	20.62
Basic Kernel and Exception Monad	25.35	45.80	47.40
Cspace	25.77	24.19	23.08
Decoding System Call	22.40	27.22	21.89
Error and Fault Message	20.36	29.38	9.07
IP	4.02	19.70	17.99
Interrupt	34.39	34.28	19.11
Kernel Object Invocation	38.11	36.57	36.18
Machine Accessor Function	25.14	25.06	25.08
Machine Operation	15.46	23.97	11.35
Retyping and Untyped Invocation	27.31	32.95	3.47
Scheduler	30.48	28.55	17.41
System Call	44.02	46.09	25.13
Threads and TCB	26.12	25.48	46.90
Toplevel ARM Definition	25.35	25.11	16.49

It is evident that the Refining method significantly improves performance for certain functionalities (e.g. “ARM VSpace and TCB”) by more than 20%, but performs poorly for others (e.g. “Retyping and Error Handling”). The reasons are as follows: First, Limited Specification Data: The model’s performance is highly influenced by its original capabilities due to the scarcity of specification data. This results in significant variations in the model’s understanding of different functionalities. Second, Over-optimization for Simple Functions: Complex logic in code requires more precise contextual understanding. During the specification generation phase, simple functions may be over-optimized, leading to deviations from the target. Consequently, the refined input may perform worse than the original. Third, Inadequacy of codeBLEU: codeBLEU does not fully represent the quality of the generated code.

To address the first two points, we believe that expanding the training dataset to enhance the model’s capabilities is essential. We plan to investigate more formal verifications of kernel specifications in future work to generate more reliable data and improve model performance. For the third point, we select specific examples to analyze the quality of code generation. Here, we still take “Access Right” as an example (Table 9).

**Table 9:** The property code of “Access Right”.

Benchmark	Generated Properties
datatype rights = AllowRead | AllowWrite | AllowGrant | AllowGrantReply	datatype access_right = ReadRight| WriteRight| GrantRight| GrantReplyRight| ReceiveRight| SyncSendRight| AsyncSendRight| ResetRight| ControlRight

It is observed that the refined specification properties for “right” define more behaviors, yet the content is correctly expressed.

## 5 Conclusion

In this paper, we introduce KerSpecGen, a framework that builds kernel specifications from requirements. Utilizing round-based dialogue and property discrimination steps, we establish a fine-grained specification for microkernel settings, progressing from specification attributes to refined specification properties. Fine-tuned large language models then generate specification code from these fine-grained specifications, ultimately synthesizing complete verification specifications. While previous approaches struggle with creating kernel specifications, ‘tree-specifier’ provides a paradigm for constructing specifications that comprehensively and clearly describe all possible specification attributes of a kernel in a tree-like graph form. The understandable relationships between specification attributes and reference specification code reduce the complexity of formal verification for complex kernels. Extensive testing shows that our method improves the accuracy of specification generation. Our ablation studies and detailed analysis demonstrate the effectiveness of each component proposed in the specifier tree.

## Appendix

See Tables 10 and 11.

**Table 10:** BLEU-4 (%) of the generated properties in Llama-3.1-70B.

Function	BLEU-4			Refining2Few	Num
	Zero-shot	Few-shot	Refining		
Accessing CSpace	47.92	44.25	58.21	13.96	13
Retyping and Untyped Invocations	29.65	56.82	55.62	–1.20	16
Threads and TCBs	0.33	4.68	24.18	19.50	15
Toplevel ARM Definitions	4.47	6.00	52.74	46.74	12
ARM Object Invocations	12.40	18.47	18.61	0.14	12
ARM VSpace Functions	0.10	0.00	6.34	6.34	37
Basic Data Structures	3.84	27.60	22.05	–5.55	28
Platform Definitions	43.60	41.76	40.84	–0.92	5
ARM Machine Instantiation	2.58	1.92	48.70	46.78	13
CSpace	17.72	19.94	13.02	–6.92	53
Abstract Specification Instantiations.txt	26.56	25.10	44.68	19.58	18
Error and Fault Messages	39.77	40.58	51.94	11.36	4
ARM-Specific Virtual-Memory Rights	41.61	43.24	40.37	–2.87	4
Accessing the ARM VSpace	18.24	43.77	35.16	–8.61	16
Access Rights	22.79	13.63	41.85	28.22	4
Accessing the Kernel Heap	9.81	54.12	51.60	–2.52	35
Basic Kernel and Exception Monads	39.87	32.06	28.38	–3.68	7
Decoding System Calls	57.54	56.19	59.20	3.01	22
IPC	54.67	58.16	53.94	–4.22	30
Machine Accessor Functions	37.67	27.38	39.00	11.62	7
IPC Cancelling	0.00	0.02	33.98	33.96	24
ARM-Specific Data Types	51.85	63.96	63.07	–0.89	30
Common, Architecture-Specific Data Types	0.00	0.91	0.91	0.00	23
Kernel Object Invocations	35.65	37.94	41.00	3.06	9
An Initial Kernel State	20.00	24.54	34.43	9.89	7
Scheduler	0.27	22.06	66.68	44.62	13
Nondeterministic State Monad with Failure	42.06	52.65	68.70	16.05	73
System Calls	24.44	29.81	2.60	–27.21	17
Machine Operations	4.83	1.15	15.09	13.94	72
Interrupts	26.25	39.28	52.51	13.23	5
Average	23.88	29.60	38.85	9.25	–

**Table 11:** BLEU-4 (%) of the generated properties in DeepseekV3.

Function	BLEU-4			Refining2Few	Num
	Zero-shot	Few-shot	Refining		
Accessing CSpace	37.06	38.85	48.02	9.17	13
Retyping and Untyped Invocations	35.19	34.50	41.83	7.33	16
Threads and TCBs	0.04	0.30	61.55	61.25	15
Toplevel ARM Definitions	41.19	38.36	45.29	6.93	12
ARM Object Invocations	24.33	36.29	33.63	–2.66	12
ARM VSpace Functions	43.90	48.94	48.77	–0.17	37
Basic Data Structures	22.05	18.61	20.51	1.90	28
Platform Definitions	38.01	32.39	31.37	–1.02	5
ARM Machine Instantiation	46.87	42.28	37.89	–4.39	13
CSpace	36.74	38.90	55.38	16.48	53
Abstract Specification Instantiations.txt	51.99	54.04	52.05	–1.99	18
Error and Fault Messages	55.90	56.59	40.36	–16.23	4
ARM-Specific Virtual-Memory Rights	35.75	28.03	25.05	–2.98	4
Accessing the ARM VSpace	71.89	71.03	67.78	–3.25	16
Access Rights	13.30	27.20	22.00	–5.20	4
Accessing the Kernel Heap	38.04	32.28	34.20	1.92	35
Basic Kernel and Exception Monads	38.11	38.16	36.32	–1.84	7
Decoding System Calls	1.05	0.40	53.02	52.62	22
IPC	42.50	37.07	55.29	18.22	30
Machine Accessor Functions	47.71	51.21	50.81	–0.40	7
IPC Cancelling	39.58	44.24	44.12	–0.12	24
ARM-Specific Data Types	43.52	42.11	40.79	–1.32	30
Common, Architecture-Specific Data Types	43.06	48.87	53.50	4.63	23
Kernel Object Invocations	51.03	50.15	57.45	7.30	9
An Initial Kernel State	33.08	25.08	23.54	–1.54	7
Scheduler	50.65	50.85	46.32	–4.53	13
Nondeterministic State Monad with Failure	50.56	58.38	57.08	–1.30	73
System Calls	61.06	61.15	63.48	2.33	17
Machine Operations	39.21	53.26	59.44	6.18	72
Interrupts	33.10	53.90	46.99	–6.91	5
Average	38.88	40.45	45.13	4.68	–

## References

[1] Klein G, Elphinstone K, Heiser G, Andronick J, Cock D, Derrin P. seL4: formal verification of an OS Kernel. In: Proceedings of the ACM SIGOPS 22nd symposium on Operating systems principles. 2009. p. 207–20.

[2] Gu R, Shao Z, Chen H, Wu XN, Kim J, Sjöberg V, et al. CertiKOS: an extensible architecture for building certified concurrent OS kernels. In: 12th USENIX Symposium on Operating Systems Design and Implementation (OSDI 16). 2016. p. 653–69.

[3] Li X, Li X, Dall C, Gu R, Nieh J, Sait Y, et al. Design and verification of the arm confidential compute architecture. In: 16th USENIX Symposium on Operating Systems Design and Implementation (OSDI 22). Carlsbad, CA: USENIX Association; 2022. p. 465–84. https://www.usenix.org/conference/osdi22/presentation/li

[4] Fox ACJ, Stockwell G, Xiong S, Becker H, Mulligan DP, Petri G, et al. A Verification Methodology for the Arm^®^ Confidential Computing Architecture: From a Secure Specification to Safe Implementations. Proc ACM Program Lang. 2023;7(OOPSLA1):376–405. doi: 10.1145/3586040

[5] Winter K, Coughlin N, Smith G. Backwards-directed information flow analysis for concurrent programs. In: 2021 IEEE 34th Computer Security Foundations Symposium (CSF). 2021. p. 1–16. 10.1109/csf51468.2021.00017

[6] Sanan D, Zhao Y, Lin SW, Yang L. CSim 2: compositional top-down verification of concurrent systems using rely-guarantee. ACM Transactions on Programming Languages and Systems. 2021;43(1):1–46.

[7] Ahmadi S, Dongol B, Griffin M. Operationally proving memory access violations in Isabelle/HOL. Science of Computer Programming. 2024;234:103088. doi: 10.1016/j.scico.2024.103088

[8] Ebalard A, Mouy P, Benadjila R. Journey to a RTE-free X. 509 parser. In: Symposium sur la sécurité des technologies de l’information et des communications (SSTIC 2019); 2019.

[9] Efremov D, Mandrykin M, Khoroshilov A. Deductive verification of unmodified Linux kernel library functions. In: Leveraging Applications of Formal Methods, Verification, Validation. Verification: 8th International Symposium and ISoLA 2018, Limassol, Cyprus, November 5-9, 2018, Proceedings, Part II. 2018. p. 216–34.

[10] Gan T, Xia B, Xue B, Zhan N, Dai L. Nonlinear Craig interpolant generation. In: International Conference on Computer Aided Verification. Springer; 2020. p. 415–38.

[11] Nelson L, Bornholt J, Gu R, Baumann A, Torlak E, Wang X. Scaling symbolic evaluation for automated verification of systems code with serval. In: Proceedings of the 27th ACM Symposium on Operating Systems Principles. 2019. p. 225–42. 10.1145/3341301.3359641

[12] Nicole O, Lemerre M, Bardin S, Rival X. No crash, no exploit: automated verification of embedded Kernels. In: 2021 IEEE 27th Real-Time and Embedded Technology and Applications Symposium (RTAS). 2021. p. 27–39. 10.1109/rtas52030.2021.00011

[13] Wu Y, Jiang AQ, Li W, Rabe M, Staats C, Jamnik M. Autoformalization with large language models. Advances in Neural Information Processing Systems. 2022;35:32353–68.

[14] Cosler M, Hahn C, Mendoza D, Schmitt F, Trippel C. nl2spec: interactively translating unstructured natural language to temporal logics with large language models. In: International Conference on Computer Aided Verification. Springer; 2023. p. 383–96.

[15] Wang H, Xin H, Zheng C, Li L, Liu Z, Cao Q. Lego-prover: neural theorem proving with growing libraries. arXiv preprint 2023. https://arxiv.org/abs/231000656

[16] Wen C, Cao J, Su J, Xu Z, Qin S, He M. Enchanting program specification synthesis by large language models using static analysis and program verification. In: International Conference on Computer Aided Verification. 2024. p. 302–28.

[17] Nigar N, Wajid A, Ajagbe SA, Adigun MO. An intelligent framework based on deep learning for online quran learning during pandemic. Applied Computational Intelligence and Soft Computing. 2023;2023:1–9. doi: 10.1155/2023/5541699

[18] Achiam J, Adler S, Agarwal S, Ahmad L, Akkaya I, Aleman FL, et al. Gpt-4 technical report. arXiv preprint 2023. doi: arXiv:230308774

[19] Li H, Dong Z, Wang S, Zhang H, Shen L, Peng X. Extracting formal specifications from documents using LLMs for automated testing. arXiv preprint 2025. doi: arXiv:250401294

[20] Ma L, Liu S, Li Y, Xie X, Bu L. Specgen: Automated generation of formal program specifications via large language models. arXiv preprint arXiv:240108807. 2024.

[21] Li M, Fang W, Zhang Q, Xie Z. SpecLLM: exploring generation and review of VLSI design specification with large language model. In: 2025 International Symposium of Electronics Design Automation (ISEDA). 2025. p. 749–55. 10.1109/iseda65950.2025.11100410

[22] Liu NF, Lin K, Hewitt J, Paranjape A, Bevilacqua M, Petroni F, et al. Lost in the middle: how language models use long contexts. Transactions of the Association for Computational Linguistics. 2024;12:157–73. doi: 10.1162/tacl_a_00638

[23] Zhan S, Lin Y, Yao Y, Zhu J. Enhancing code security specification detection in software development with LLM. In: 2025 7th International Conference on Information Science, Electrical and Automation Engineering (ISEAE). IEEE; 2025. p. 1079–83.

[24] Krishna M, Gaur B, Verma A, Jalote P. Using LLMs in software requirements specifications: an empirical evaluation. In: 2024 IEEE 32nd International Requirements Engineering Conference (RE). 2024. p. 475–83. 10.1109/re59067.2024.00056

[25] ISWC D, et al. The semantic web-iswc 2017 : 16th international semantic web conference, Vienna, Austria, October 21-25, 2017: proceedings. In: Vienna, Austria, 2017.

[26] Zhang Y, Fu-Tai Z. Detection method of malicious domain name based on knowledge map. Communications Technology. 2020;53(1):168–73.

[27] Du D, Ren X, Wu Y, Chen J, Ye W, Sun J, et al. Refining traceability links between vulnerability, software component in a vulnerability knowledge graph. In: Web Engineering: 18th International Conference and ICWE 2018, Cáceres, Spain, June 5-8, 2018, Proceedings 18. Springer; 2018. p. 33–49.

[28] Qin S, Chow K. Automatic analysis, reasoning based on vulnerability knowledge graph. In: Cyberspace Data, Intelligence,, Cyber-Living, Syndrome and Health: International 2019 Cyberspace Congress, CyberDI and CyberLife, Beijing, China, December 16–18, 2019, Proceedings, Part I. 2019. p. 3–19.

[29] Xiao H, Xing Z, Li X, Guo H. Embedding, predicting software security entity relationships: a knowledge graph based approach. In: Neural Information Processing: 26th International Conference and ICONIP 2019, Sydney, NSW, Australia, December 12–15, 2019, Proceedings, Part III. 2019. p. 50–63.

[30] Ren X, Ye X, Xing Z, Xia X, Xu X, Zhu L, et al. API-misuse detection driven by fine-grained API-constraint knowledge graph. In: Proceedings of the 35th IEEE/ACM International Conference on Automated Software Engineering. 2020. p. 461–72. 10.1145/3324884.3416551

[31] Ling C-Y, Zou Y-Z, Lin Z-Q, Xie B. Graph embedding based API graph search and recommendation. J Comput Sci Technol. 2019;34(5):993–1006. doi: 10.1007/s11390-019-1956-2

[32] Wang L, Sun X, Wang J, Duan Y, Li B. Construct bug knowledge graph for bug resolution. In: 2017 IEEE/ACM 39th International Conference on Software Engineering Companion (ICSE-C). 2017. p. 189–91. 10.1109/icse-c.2017.102

[33] Zhang S, Liu X, Xu B, Cai L, Hu Y. Construction of a cloud scenario knowledge graph for cloud service market. In: 2020 IEEE 11th International Conference on Software Engineering and Service Science (ICSESS). 2020. p. 503–6. 10.1109/icsess49938.2020.9237681

[34] Luo C, Liu X, Zhang K, Chang Q. A recommendation system for cloud services based on knowledge graph. In: 2018 IEEE 9th International Conference on Software Engineering and Service Science (ICSESS). 2018. p. 1–4. 10.1109/icsess.2018.8663716

[35] Andronick J, Bourke T, Derrin P, Elphinstone K, Greenaway D, Klein G. Abstract formal specification of the seL4/ARMv6 API. 2014.

[36] Parnami A, Lee M. Learning from few examples: a summary of approaches to few-shot learning. arXiv preprint 2022. doi: arXiv:220304291

[37] Piantadosi ST, Tily H, Gibson E. The communicative function of ambiguity in language. Cognition. 2012;122(3):280–91. doi: 10.1016/j.cognition.2011.10.004 22192697

[38] Zhang Z, Wang C, Wang Y, Shi E, Ma Y, Zhong W, et al. LLM hallucinations in practical code generation: phenomena, mechanism, and mitigation. Proc ACM Softw Eng. 2025;2(ISSTA):481–503. doi: 10.1145/3728894

[39] Cherem S, Chilimbi T, Gulwani S. Inferring locks for atomic sections. SIGPLAN Not. 2008;43(6):304–15. doi: 10.1145/1379022.1375619

[40] Arslan M, Ghanem H, Munawar S, Cruz C. A survey on RAG with LLMs. Procedia Computer Science. 2024;246:3781–90. doi: 10.1016/j.procs.2024.09.178

[41] Wang W, Zheng VW, Yu H, Miao C. A survey of zero-shot learning: settings, methods, and applications. ACM Transactions on Intelligent Systems and Technology (TIST). 2019;10(2):1–37.

[42] Papineni K, Roukos S, Ward T, Zhu WJ. Bleu: a method for automatic evaluation of machine translation. In: Proceedings of the 40th annual meeting of the Association for Computational Linguistics; 2002. p. 311–8.

[43] Heiser G. The sel4 microkernel–an introduction. The seL4 Foundation. 2020;1.

[44] Lin CY. Rouge: a package for automatic evaluation of summaries. In: Text summarization branches out; 2004. p. 74–81.

[45] Ren S, Guo D, Lu S, Zhou L, Liu S, Tang D, et al. Codebleu: a method for automatic evaluation of code synthesis. arXiv preprint 2020. https://arxiv.org/abs/2009.10297

[46] Bird S. NLTK: the natural language toolkit. In: Proceedings of the COLING/ACL 2006 interactive presentation sessions. 2006. p. 69–72.

[47] Neamtiu I, Foster JS, Hicks M. Understanding source code evolution using abstract syntax tree matching. In: Proceedings of the 2005 international workshop on mining software repositories. 2005. p. 1–5.

